# Supplementary material for: Near-Infrared MAO A Inhibitor (NMI) Outperformed FDA-Approved Chemotherapeutic Agents in Brain and Other Cancers: A Bioinformatic Analysis of NCI60 Screening Data
Source: Brain Sci. 2021 Oct 4;11(10):1318. doi: 10.3390/brainsci11101318 (PMC8534240; doi:10.3390/brainsci11101318)
Supplement: Supplementary file 1 [file brainsci-11-01318-s001.zip › brainsci-1296779-SupplementaryMaterial.pdf]

Supplementary Data

A

CNS Cancer		
**Drug**	**Indication**	**Mechanism of Action**
Temozolomide	glioblastoma concomitantly with radiotherapy and refractory anaplastic astrocytoma	DNA and RNA Alkylation
Lomustine	primary and metastatic brain tumors	DNA and RNA Alkylation
Carmustine	brain tumors glioblastoma, brainstem glioma, medulloblastoma, astrocytoma, ependymoma, and metastatic brain tumors	DNA and RNA alkylation; Enzymatic Carbamoylation
Everolimus	subependymal giant cell astrocytoma (SEGA) that requires therapeutic intervention	mTOR inhibitor; HIF inhibitor

B

Prostate Cancer		
**Drug**	**Indication**	**Mechanism of Action**
Rucaparib	metastatic Castration-Resistant Prostate Cancer (mCRPC)	PARP Inhibitor
Enzalutamide	mCRPC and metastatic Castration-Sensitive Prostate Cancer (mCSPC)	Androgen Receptor Inhibitor
Abiraterone Acetate	mCRPC and mCSPC	CYP17 Inhibitor
Cabazitaxel	mCRPC	Antimicrotubular Antineoplastic Agent
Docetaxel	mCRPC	Antimicrotubular Antineoplastic Agent
Leuprolide Acetate	palliative treatment of advanced prostate cancer	GnRH Agonist
Mitoxantrone	advanced prostate cancer not responding to hormone treatment	Topoisomerase Inhibitor
Olaparib	mCRPC	PARP Inhibitor

C

Non-Small Cell Lung Cancer		
**Drug**	**Indication**	**Mechanism of Action**
Alectinib	ALK-positive metastatic NSCLC	ALK and RET Inhibitor
Pemetrexed	locally advanced or metastatic, non-squamous NSCLC	Antimetabolite
Carboplatin	chemotherapeutic treatment for NSCLC	Alkylating Agent
Crizotinib	ALK or ROS1-positive metastatic NSCLC	ALK and ROS1 Inhibitor
Docetaxel	locally advanced or metastatic NSCLC after failure of prior platinum-based chemotherapy	Taxoid
Doxorubicin	chemotherapeutic treatment for NSCLC	Anthracycline Antibiotic
Erlotinib	EGFR gene mutated metastatic NSCLC	EGFR Tyrosine Kinase Inhibitor
Everolimus	unresectable, locally advanced, or metastatic NSCLC	mTOR Kinase Inhibitor
Gefitinib	EGFR gene mutated metastatic NSCLC; in combination with Everolimus	EGFR Tyrosine Kinase Inhibitor
Lorlatinib	ALK-positive metastatic NSCLC	ALK and ROS1 Inhibitor
Mechlorethamine	NSCLC	Alkylating Agent
Trametinib	in combination with dabrafenib for BRAF V600E mutated metastatic NSCLC	MEK Kinase Inhibitor
Methotrexate	NSCLC	Folate Analog Metabolic Inhibitor
Paclitaxel	chemotherapeutic treatment for NSCLC	Taxane; Plant Alkaloid
Vinorelbine	alone or in combination with Cisplatin for locally advanced or metastatic NSCLC	Plant Alkaloid

Table S1 The indication and mechanism of FDA-approved anticancer drugs for (A)CNS cancer**, (B)** prostate cancer **(C)** NSCLC. Literature review of indication, mechanism of FDA-approved CNS, prostate, NSCLC cancers.

A

	Cell Lines	NMI	Temozolomide	Lomustine	Carmustine	Everolimus	Max	Min	Max-Min
**GI50 (μM)**	**SF-268**	1.820	100.000	20.900	31.600	0.269	100.000	0.269	99.731
Min-max normalization		0.016	1.000	0.207	0.314	0.000	(GI50 - Min) / (Max - Min)		
		e.g. Min-max normalization of GI50 of NMI: (1.820-0.269)/99.731=0.016							
**GI50 (μM)**	**SF-295**	1.910	100.000	91.200	39.800	0.010	100.000	0.010	99.990
Min-max normalization		0.019	1.000	0.912	0.398	0.000	(GI50 - Min) / (Max - Min)		
**GI50 (μM)**	**SF-539**	1.200	100.000	16.600	31.600	0.010	100.000	0.010	99.990
Min-max normalization		0.012	1.000	0.166	0.316	0.000	(GI50 - Min) / (Max - Min)		
**GI50 (μM)**	**SNB-19**	1.120	100.000	100.000	63.100	0.257	100.000	0.257	99.743
Min-max normalization		0.009	1.000	1.000	0.630	0.000	(GI50 - Min) / (Max - Min)		
**GI50 (μM)**	**SNB-75**	1.290	63.000	23.400	50.100	0.010	63.000	0.010	62.990
Min-max normalization		0.020	1.000	0.371	0.795	0.000	(GI50 - Min) / (Max - Min)		
**GI50 (μM)**	**U251**	0.740	100.000	15.800	31.600	0.011	100.000	0.011	99.989
Min-max normalization		0.007	1.000	0.158	0.316	0.000	(GI50 - Min) / (Max - Min)		
**TGI (μM)**	**SF-268**	6.760	100.000	57.500	100.000	20.900	100.000	6.760	93.240
Min-max normalization		0.000	1.000	0.544	1.000	0.152	(TGI - Min) / (Max - Min)		
**TGI (μM)**	**SF-295**	4.070	100.000	100.000	251.000	17.300	251.000	4.070	246.930
Min-max normalization		0.000	0.388	0.388	1.000	0.054	(TGI - Min) / (Max - Min)		
**TGI (μM)**	**SF-539**	2.690	100.000	33.900	100.000	17.300	100.000	2.690	97.310
Min-max normalization		0.000	1.000	0.321	1.000	0.150	(TGI - Min) / (Max - Min)		
**TGI (μM)**	**SNB-19**	3.630	100.000	100.000	251.000	19.500	251.000	3.630	247.370
Min-max normalization		0.000	0.390	0.390	1.000	0.064	(TGI - Min) / (Max - Min)		
**TGI (μM)**	**SNB-75**	3.890	100.000	64.600	126.000	14.100	126.000	3.890	122.110
Min-max normalization		0.000	0.787	0.497	1.000	0.084	(TGI - Min) / (Max - Min)		
**TGI (μM)**	**U251**	2.090	100.000	51.300	63.100	18.200	100.000	2.090	97.910
Min-max normalization		0.000	1.000	0.503	0.623	0.165	(TGI - Min) / (Max - Min)		
**LC50 (μM)**	**SF-268**	100.000	100.000	100.000	251.000	53.700	251.000	53.700	197.300
Min-max normalization		0.235	0.235	0.235	1.000	0.000	(LC50 - Min) / (Max - Min)		
**LC50 (μM)**	**SF-295**	8.710	100.000	100.000	251.000	63.100	251.000	8.710	242.290
Min-max normalization		0.000	0.377	0.377	1.000	0.224	(LC50 - Min) / (Max - Min)		
**LC50 (μM)**	**SF-539**	6.030	100.000	70.800	251.000	41.700	251.000	6.030	244.970
Min-max normalization		0.000	0.384	0.264	1.000	0.146	(LC50 - Min) / (Max - Min)		
**LC50 (μM)**	**SNB-19**	28.200	100.000	100.000	251.000	44.700	251.000	28.200	222.800
Min-max normalization		0.000	0.322	0.322	1.000	0.074	(LC50 - Min) / (Max - Min)		
**LC50 (μM)**	**SNB-75**	13.800	100.000	100.000	251.000	38.900	251.000	13.800	237.200
Min-max normalization		0.000	0.363	0.363	1.000	0.106	(LC50 - Min) / (Max - Min)		
**LC50 (μM)**	**U251**	5.010	100.000	100.000	126.000	42.700	126.000	5.010	120.990
Min-max normalization		0.000	0.785	0.785	1.000	0.312	(LC50 - Min) / (Max - Min)		
**Cumulative Score**	**SF-268**	0.250	2.235	0.986	2.314	0.152	GI50+TGI+LC50 Min-max normalization		
	**SF-295**	0.019	1.765	1.677	2.398	0.278			
	**SF-539**	0.012	2.384	0.751	2.316	0.296			
	**SNB-19**	0.009	1.712	1.712	2.630	0.138			
	**SNB-75**	0.020	2.150	1.232	2.795	0.189			
	**U251**	0.007	2.785	1.446	1.939	0.476			
		e.g. Cumulative score of min-max normalization of GI50, TGI and LC50 of NMI: 0.016+0.000+0.235=0.250							

B

	Cell Lines	NMI	Rucaparib	Enzalutamide	Abiraterone Acetate	Cabazitaxel	Docetaxel	Leuprolide Acetate	Mitoxantrone	Olaparib	Max	Min	Max-Min
**GI50 (μM)**	**PC-3**	1.700	23.400	20.000	3.980	0.010	0.010	100.000	0.141	51.286	100.000	0.010	99.990
Min-max normalization		0.017	0.234	0.200	0.040	0.000	0.000	1.000	0.001	0.513	(GI50 - Min) / (Max - Min)		
		e.g. Min-max normalization of GI50 of NMI: (1.700-0.010)/99.99 = 0.017											
**GI50 (μM)**	**DU-145**	3.020	18.200	31.600	20.000	0.010	0.010	100.000	0.018	100.000	100.000	0.010	99.990
Min-max normalization		0.030	0.182	0.316	0.200	0.000	0.000	1.000	0.000	1.000	(GI50 - Min) / (Max - Min)		
**TGI (μM)**	**PC-3**	3.980	100.000	100.000	100.000	79.400	100.000	100.000	1.445	100.000	100.000	1.445	98.555
Min-max normalization		0.026	1.000	1.000	1.000	0.791	1.000	1.000	0.000	1.000	(TGI - Min) / (Max - Min)		
**TGI (μM)**	**DU-145**	11.000	36.300	100.000	100.000	0.010	77.625	100.000	0.468	100.000	100.000	0.010	99.990
Min-max normalization		0.110	0.363	1.000	1.000	0.000	0.776	1.000	0.005	1.000	(TGI - Min) / (Max - Min)		
**LC50 (μM)**	**PC-3**	9.550	100.000	100.000	100.000	100.000	100.000	100.000	10.471	100.000	100.000	9.550	90.450
Min-max normalization		0.000	1.000	1.000	1.000	1.000	1.000	1.000	0.010	1.000	(LC50 - Min) / (Max - Min)		
**LC50 (μM)**	**DU-145**	34.700	72.400	100.000	100.000	100.000	100.000	100.000	11.482	100.000	100.000	11.482	88.518
Min-max normalization		0.262	0.688	1.000	1.000	1.000	1.000	1.000	0.000	1.000	(LC50 - Min) / (Max - Min)		
**Cumulative Score**	**PC-3**	0.043	2.234	2.200	2.040	1.791	2.000	3.000	0.011	2.513	GI50+TGI+LC50 Min-max normalization		
	**DU-145**	0.402	1.233	2.316	2.200	1.000	1.776	3.000	0.005	3.000			
		e.g. Cumulative score of min-max normalization of GI50, TGI and LC50 of NMI: 0.017+0.026+0.000=0.043											

(Continued)

(Continued)

C

	Cell Lines	NMI	Erlotinib	Trametinib	Alectinib	Pemetrexed	Carboplatin	Crizotinib	Docetaxel	Doxorubicin	Everolimus	Gefitinib	Lorlatinib	Mechlorethamine	Methotrexate	Paclitaxel	Vinorelbine	Max	Min	Max-Min
GI50 (μM)	A549/ATCC	3.220	9.120	0.030	1.350	0.590	100.000	1.170	0.010	0.063	0.010	7.590	25.119	2.818	0.010	0.011	0.019	100.000	0.010	99.990
Min-max normalization	A549/ATCC	0.032	0.091	0.000	0.013	0.006	1.000	0.012	0.000	0.001	0.000	0.076	0.251	0.028	0.000	0.000	0.000	(GI50 - Min) / (Max - Min)		
		e.g. Min-max normalization of GI50 of NMI: (3.220-0.010)/99.990=0.032																		
GI50 (μM)	EKVX	2.420	0.191	0.013	1.550	100.000	100.000	5.130	3.720	0.420	0.010	0.045	16.218	6.026	10.000	0.977	0.010	100.000	0.010	99.990
Min-max normalization	EKVX	0.024	0.002	0.000	0.015	1.000	1.000	0.051	0.037	0.004	0.000	0.000	0.162	0.060	0.100	0.010	0.000	(GI50 - Min) / (Max - Min)		
GI50 (μM)	HOP-62	2.100	15.100	0.251	0.850	0.720	100.000	1.910	0.010	0.068	0.010	10.200	22.909	5.248	0.016	0.031	0.015	100.000	0.010	99.990
Min-max normalization	HOP-62	0.021	0.151	0.002	0.008	0.007	1.000	0.019	0.000	0.001	0.000	0.102	0.229	0.052	0.000	0.000	0.000	(GI50 - Min) / (Max - Min)		
GI50 (μM)	HOP-92	1.570	4.270	0.010	0.390	100.000	100.000	0.560	9.120	0.100	0.020	7.410	2.512	0.372	7.943	0.525	0.047	100.000	0.010	99.990
Min-max normalization	HOP-92	0.016	0.043	0.000	0.004	1.000	1.000	0.006	0.091	0.001	0.000	0.074	0.025	0.004	0.079	0.005	0.000	(GI50 - Min) / (Max - Min)		
GI50 (μM)	NCI-H226	1.720	42.700	4.370	1.740	100.000	100.000	1.820	0.360	0.050	0.350	15.500	40.738	8.318	2.344	1.047	0.030	100.000	0.030	99.970
Min-max normalization	NCI-H226	0.017	0.427	0.043	0.017	1.000	1.000	0.018	0.003	0.000	0.003	0.155	0.407	0.083	0.023	0.010	0.000	(GI50 - Min) / (Max - Min)		
GI50 (μM)	NCI-H23	1.940	28.200	0.010	2.820	100.000	100.000	2.090	0.010	0.150	0.320	17.000	58.884	1.318	0.087	0.012	0.019	100.000	0.010	99.990
Min-max normalization	NCI-H23	0.019	0.282	0.000	0.028	1.000	1.000	0.021	0.000	0.001	0.003	0.170	0.589	0.013	0.001	0.000	0.000	(GI50 - Min) / (Max - Min)		
GI50 (μM)	NCI-H322M	3.010	0.098	0.012	2.340	100.000	100.000	5.250	0.010	0.540	0.220	0.085	33.113	15.849	0.427	0.009	0.010	100.000	0.009	99.991
Min-max normalization	NCI-H322M	0.030	0.001	0.000	0.023	1.000	1.000	0.052	0.000	0.005	0.002	0.001	0.331	0.158	0.004	0.000	0.000	(GI50 - Min) / (Max - Min)		
GI50 (μM)	NCI-H460	1.770	5.890	3.160	1.480	0.280	100.000	0.890	0.010	0.017	0.150	6.610	28.184	0.129	0.006	0.007	0.010	100.000	0.006	99.995
Min-max normalization	NCI-H460	0.018	0.059	0.032	0.015	0.003	1.000	0.009	0.000	0.000	0.001	0.066	0.282	0.001	0.000	0.000	0.000	(GI50 - Min) / (Max - Min)		
GI50 (μM)	NCI-H522	1.160	0.955	3.800	1.170	18.200	100.000	1.510	0.010	0.029	0.160	6.760	57.544	0.871	1.445	0.004	0.023	100.000	0.004	99.996
Min-max normalization	NCI-H522	0.012	0.010	0.038	0.012	0.182	1.000	0.015	0.000	0.000	0.002	0.068	0.575	0.009	0.014	0.000	0.000	(GI50 - Min) / (Max - Min)		
TGI (μM)	A549/ATCC	13.200	100.000	43.700	3.090	100.000	100.000	11.200	81.300	0.290	22.400	21.900	100.000	32.359	10.000	70.795	33.884	100.000	0.290	99.710
Min-max normalization	A549/ATCC	0.129	1.000	0.435	0.028	1.000	1.000	0.109	0.812	0.000	0.222	0.217	1.000	0.322	0.097	0.707	0.337	(TGI - Min) / (Max - Min)		
TGI (μM)	EKVX	12.800	100.000	70.800	6.920	100.000	100.000	18.200	100.000	5.500	21.400	17.400	100.000	26.303	10.000	85.114	18.621	100.000	5.500	94.500
Min-max normalization	EKVX	0.077	1.000	0.691	0.015	1.000	1.000	0.134	1.000	0.000	0.168	0.126	1.000	0.220	0.048	0.842	0.139	(TGI - Min) / (Max - Min)		
TGI (μM)	HOP-62	5.720	56.200	51.300	2.630	100.000	100.000	7.590	3.800	2.000	22.400	35.500	100.000	31.623	5.495	39.811	24.547	100.000	2.000	98.000
Min-max normalization	HOP-62	0.038	0.553	0.503	0.006	1.000	1.000	0.057	0.018	0.000	0.208	0.342	1.000	0.302	0.036	0.386	0.230	(TGI - Min) / (Max - Min)		
TGI (μM)	HOP-92	3.530	61.700	0.010	3.550	100.000	100.000	3.310	56.200	2.000	20.000	23.400	53.703	3.162	10.000	61.660	0.550	100.000	0.010	99.990
Min-max normalization	HOP-92	0.035	0.617	0.000	0.035	1.000	1.000	0.033	0.562	0.020	0.200	0.234	0.537	0.032	0.100	0.617	0.005	(TGI - Min) / (Max - Min)		
TGI (μM)	NCI-H226	4.110	100.000	47.900	4.370	100.000	100.000	7.240	63.100	0.250	23.400	33.100	100.000	39.811	10.000	19.055	36.308	100.000	0.250	99.750
Min-max normalization	NCI-H226	0.039	1.000	0.478	0.041	1.000	1.000	0.070	0.630	0.000	0.232	0.329	1.000	0.397	0.098	0.189	0.361	(TGI - Min) / (Max - Min)		
TGI (μM)	NCI-H23	5.090	100.000	19.500	38.900	100.000	100.000	15.500	75.900	0.910	22.900	46.800	100.000	8.511	10.000	72.444	41.687	100.000	0.910	99.090
Min-max normalization	NCI-H23	0.042	1.000	0.188	0.383	1.000	1.000	0.147	0.757	0.000	0.222	0.463	1.000	0.077	0.092	0.722	0.412	(TGI - Min) / (Max - Min)		
TGI (μM)	NCI-H322M	10.900	13.800	47.900	10.470	100.000	100.000	18.600	93.300	5.010	0.480	17.000	100.000	45.709	10.000	79.433	25.704	100.000	0.480	99.520
Min-max normalization	NCI-H322M	0.105	0.134	0.476	0.100	1.000	1.000	0.182	0.933	0.046	0.000	0.166	1.000	0.454	0.096	0.793	0.253	(TGI - Min) / (Max - Min)		
TGI (μM)	NCI-H460	5.010	91.200	93.300	4.070	100.000	100.000	5.620	40.700	1.290	19.500	20.000	100.000	2.754	10.000	40.738	0.447	100.000	0.447	99.553
Min-max normalization	NCI-H460	0.046	0.912	0.933	0.036	1.000	1.000	0.052	0.404	0.008	0.191	0.196	1.000	0.023	0.096	0.405	0.000	(TGI - Min) / (Max - Min)		
TGI (μM)	NCI-H522	3.340	22.400	21.900	3.800	100.000	100.000	4.170	0.100	0.160	17.800	38.900	100.000	2.399	10.000	0.036	31.623	100.000	0.036	99.964
Min-max normalization	NCI-H522	0.033	0.224	0.219	0.038	1.000	1.000	0.041	0.001	0.001	0.178	0.389	1.000	0.024	0.100	0.000	0.316	(TGI - Min) / (Max - Min)		
LC50 (μM)	A549/ATCC	38.900	100.000	89.100	6.920	100.000	100.000	36.300	100.000	100.000	70.800	46.800	100.000	100.000	10.000	91.201	66.069	100.000	6.920	93.080
Min-max normalization	A549/ATCC	0.344	1.000	0.883	0.000	1.000	1.000	0.316	1.000	1.000	0.686	0.428	1.000	1.000	0.033	0.905	0.635	(LC50 - Min) / (Max - Min)		
LC50 (μM)	EKVX	100.000	100.000	100.000	100.000	100.000	100.000	43.700	100.000	47.900	85.100	51.300	100.000	83.176	10.000	100.000	63.096	100.000	10.000	90.000
Min-max normalization	EKVX	1.000	1.000	1.000	1.000	1.000	1.000	0.374	1.000	0.421	0.834	0.459	1.000	0.813	0.000	1.000	0.590	(LC50 - Min) / (Max - Min)		
LC50 (μM)	HOP-62	22.500	100.000	100.000	6.460	100.000	100.000	28.800	93.300	67.600	55.000	74.100	100.000	100.000	10.000	85.114	85.114	100.000	6.460	93.540
Min-max normalization	HOP-62	0.171	1.000	1.000	0.000	1.000	1.000	0.239	0.928	0.654	0.519	0.723	1.000	1.000	0.038	0.841	0.841	(LC50 - Min) / (Max - Min)		
LC50 (μM)	HOP-92	7.890	100.000	43.700	40.700	100.000	100.000	17.700	100.000	42.700	53.700	49.000	100.000	21.878	10.000	100.000	42.658	100.000	7.890	92.110
Min-max normalization	HOP-92	0.000	1.000	0.389	0.356	1.000	1.000	0.107	1.000	0.378	0.497	0.446	1.000	0.152	0.023	1.000	0.377	(LC50 - Min) / (Max - Min)		
LC50 (μM)	NCI-H226	9.850	100.000	100.000	14.500	100.000	100.000	20.900	100.000	6.460	64.600	70.800	100.000	100.000	10.000	81.283	81.283	100.000	6.460	93.540
Min-max normalization	NCI-H226	0.036	1.000	1.000	0.086	1.000	1.000	0.154	1.000	0.000	0.622	0.688	1.000	1.000	0.038	0.800	0.800	(LC50 - Min) / (Max - Min)		
LC50 (μM)	NCI-H23	100.000	100.000	100.000	100.000	100.000	100.000	46.800	100.000	13.180	56.200	95.500	100.000	43.652	10.000	95.499	100.000	100.000	10.000	90.000
Min-max normalization	NCI-H23	1.000	1.000	1.000	1.000	1.000	1.000	0.409	1.000	0.035	0.513	0.950	1.000	0.374	0.000	0.950	1.000	(LC50 - Min) / (Max - Min)		
LC50 (μM)	NCI-H322M	39.500	75.900	100.000	91.200	100.000	100.000	46.800	100.000	67.600	39.800	58.900	100.000	100.000	10.000	100.000	69.183	100.000	10.000	90.000
Min-max normalization	NCI-H322M	0.328	0.732	1.000	0.902	1.000	1.000	0.409	1.000	0.640	0.331	0.543	1.000	1.000	0.000	1.000	0.658	(LC50 - Min) / (Max - Min)		
LC50 (μM)	NCI-H460	47.100	100.000	100.000	12.000	100.000	100.000	27.500	100.000	51.300	50.100	45.700	100.000	22.909	10.000	93.325	35.481	100.000	10.000	90.000
Min-max normalization	NCI-H460	0.412	1.000	1.000	0.022	1.000	1.000	0.194	1.000	0.459	0.446	0.397	1.000	0.143	0.000	0.926	0.283	(LC50 - Min) / (Max - Min)		
LC50 (μM)	NCI-H522	9.660	97.700	100.000	28.800	100.000	100.000	12.600	85.100	2.820	47.900	87.100	100.000	6.026	10.000	74.131	70.795	100.000	2.820	97.180
Min-max normalization	NCI-H522	0.070	0.976	1.000	0.267	1.000	1.000	0.101	0.847	0.000	0.464	0.867	1.000	0.033	0.074	0.734	0.699	(LC50 - Min) / (Max - Min)		
Cumulative Score	A549/ATCC	0.505	2.091	1.318	0.041	2.006	3.000	0.437	1.812	1.001	0.908	0.721	2.251	1.350	0.130	1.613	0.972	GI50+TGI+LC50 Min-max normalization		
Cumulative Score	EKVX	1.101	2.002	1.691	1.030	3.000	3.000	0.560	2.037	0.425	1.003	0.585	2.162	1.093	0.148	1.852	0.729			
Cumulative Score	HOP-62	0.230	1.704	1.505	0.015	2.007	3.000	0.315	0.947	0.654	0.727	1.167	2.229	1.355	0.074	1.227	1.071			
Cumulative Score	HOP-92	0.051	1.660	0.389	0.395	3.000	3.000	0.145	1.653	0.399	0.697	0.754	1.562	0.187	0.202	1.622	0.383			
Cumulative Score	NCI-H226	0.092	2.427	1.521	0.144	3.000	3.000	0.242	1.633	0.000	0.857	1.172	2.407	1.480	0.159	0.999	1.161			
Cumulative Score	NCI-H23	1.061	2.282	1.188	1.411	3.000	3.000	0.577	1.757	0.037	0.738	1.583	2.589	0.464	0.093	1.672	1.412			
Cumulative Score	NCI-H322M	0.462	0.867	1.477	1.026	3.000	3.000	0.643	1.933	0.691	0.333	0.710	2.331	1.613	0.100	1.793	0.911			
Cumulative Score	NCI-H460	0.476	1.970	1.964	0.073	2.003	3.000	0.255	1.404	0.467	0.638	0.659	2.282	0.168	0.096	1.331	0.283			
Cumulative Score	NCI-H522	0.115	1.210	1.257	0.317	2.182	3.000	0.157	0.847	0.001	0.643	1.324	2.575	0.065	0.188	0.734	1.016			
		e.g. Cumulative score of min-max normalization of GI50, TGI and LC50 of NMI: 0.032+0.129+0.344=0.505																		

Table S2 The min-max normalization value and the cumulative score of GI_{50}, TGI, and LC_{50} in different (A)CNS, (B) prostate, (C)NSCLC cancer cell lines for NMI and FDA-approved CNS cancer drugs. Table of the min-max normalization values and the cumulative scores of Figure 4 and Figure S3. Step-by-step calculation was provided. NMI is more potent than FDA-approved CNS, prostate, NSCLC cancer drugs.

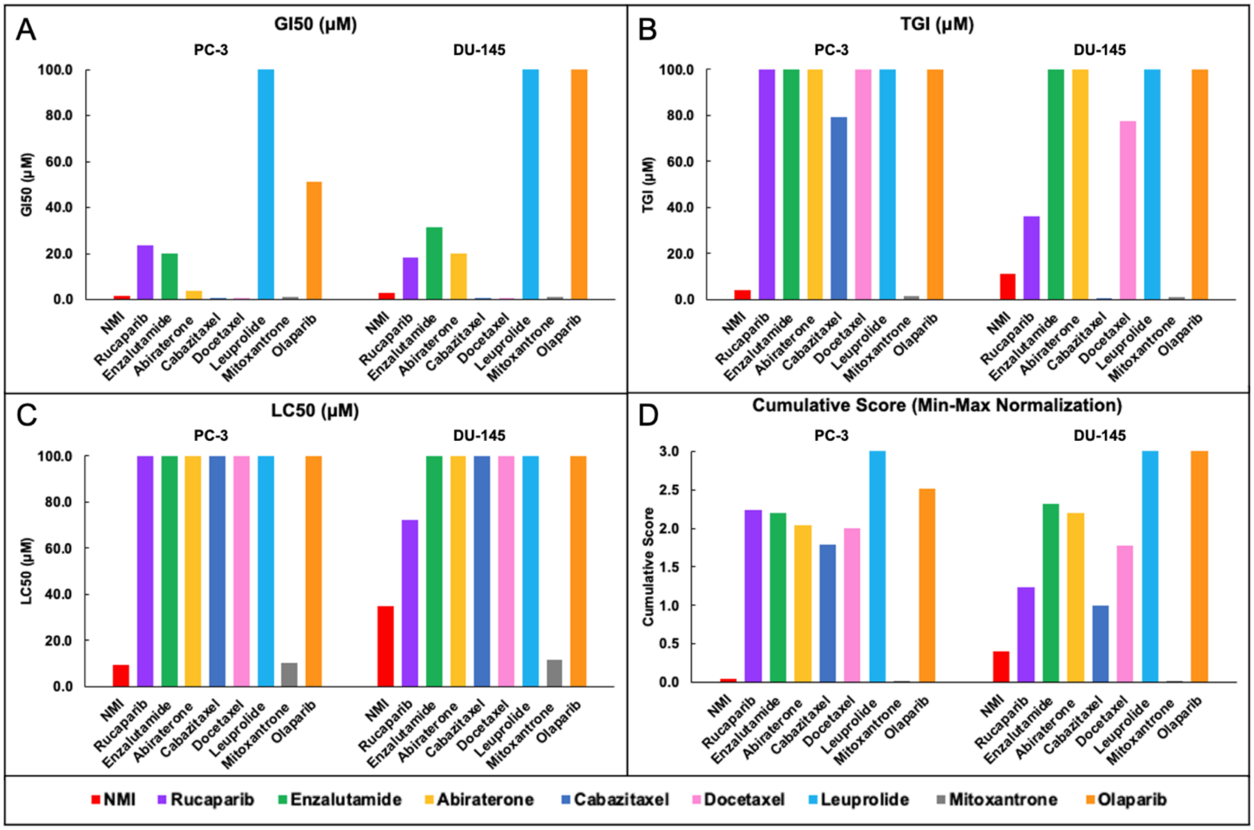

Figure S1 The comparison of (A)GI$_{50}$, (B)TGL, (C) LC$_{50}$, (D)cumulative score of NMI to FDA-approved prostate cancer drugs. NMI shows higher potency to prostate cancer cell lines than most FDA-approved prostate cancer drugs. Y-axis indicates concentration of GI$_{50}$, TGI, LC$_{50}$ (0 µM to 100 µM), and cumulative score (0.0 to 3.0). Drugs are color-coded: NMI (■); Rucaparib (■), Enzalutamide (■); Abiraterone (■); Cabazitaxel (■); Docetaxel (■); Leuprolide (■); Mitoxantrone (■); Olaparib (■).

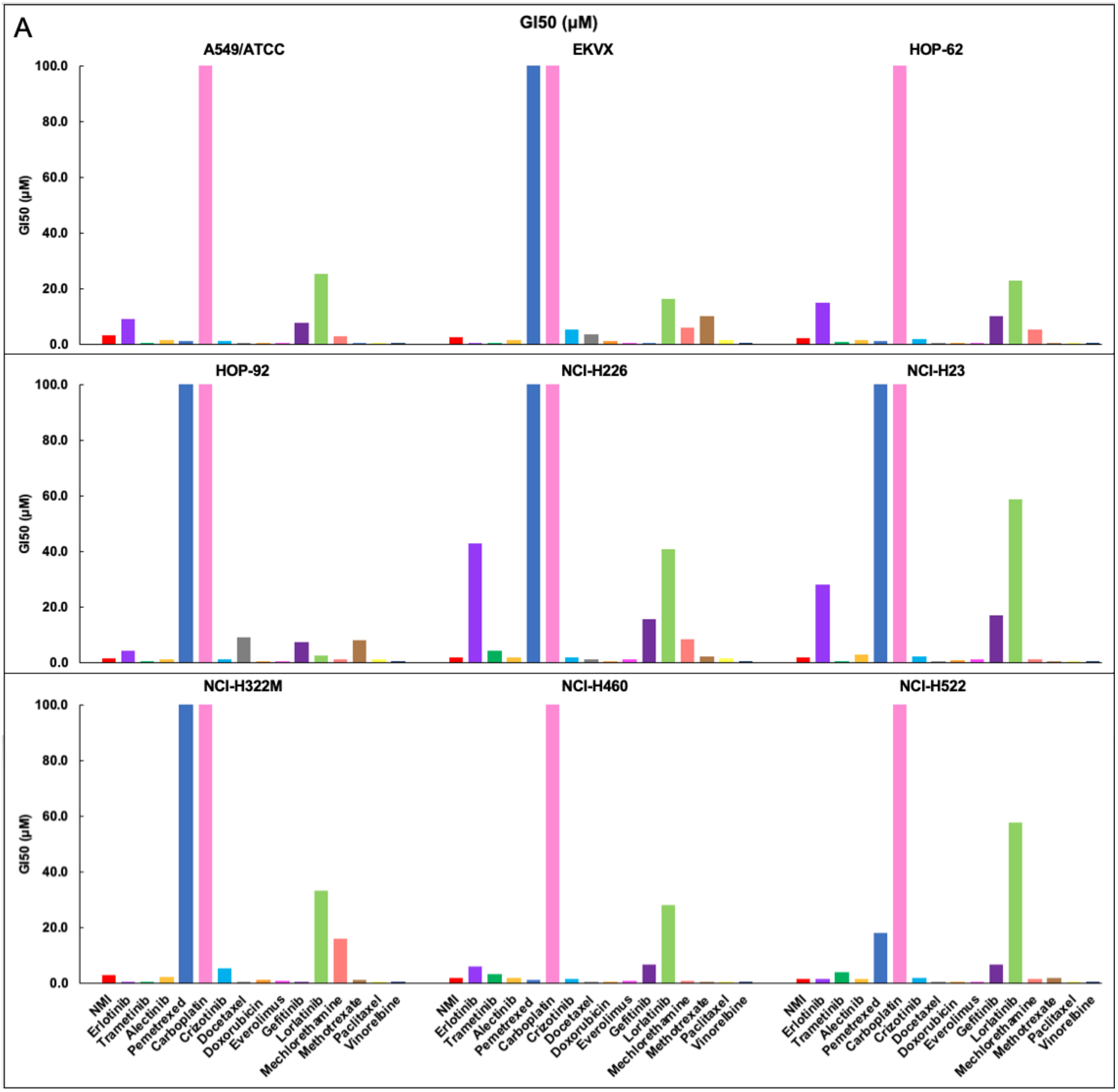
A
GI50 (μM)
A549/ATCC
EKVX
HOP-62
HOP-92
NCI-H226
NCI-H23
NCI-H322M
NCI-H460
NCI-H522
GI50 (μM)
100.0
80.0
60.0
40.0
20.0
0.0
NMI
Erlotinib
Trametinib
Alectinib
Pemetrexed
Carboplatin
Crizotinib
Docetaxel
Doxorubicin
Everolimus
Gefitinib
Lorlatinib
Mechlorethamine
Methotrexate
Paclitaxel
Vinorelbine

(Continued)

(Continued)

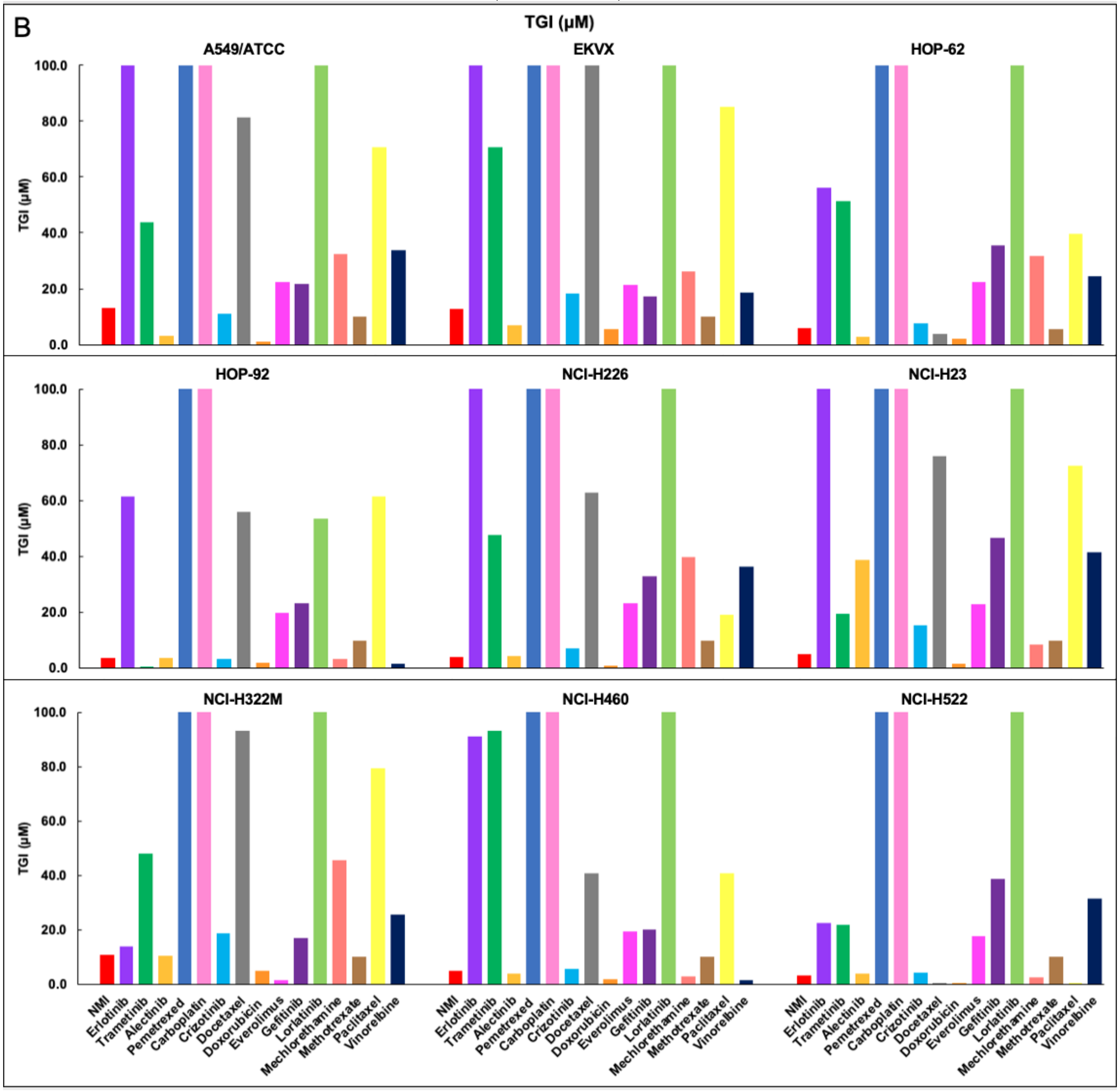

(Continued)

(Continued)

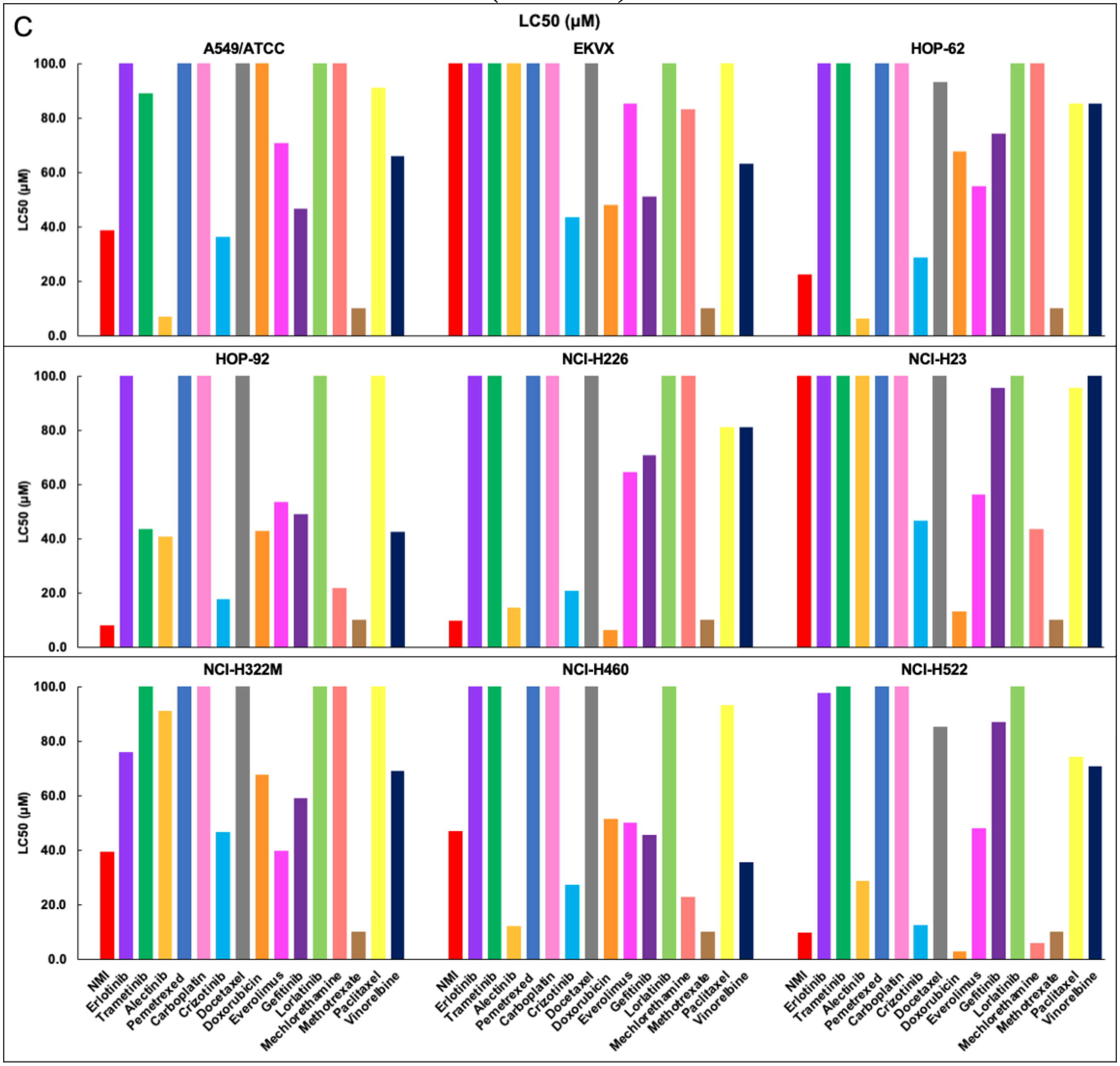

(Continued)

(Continued)

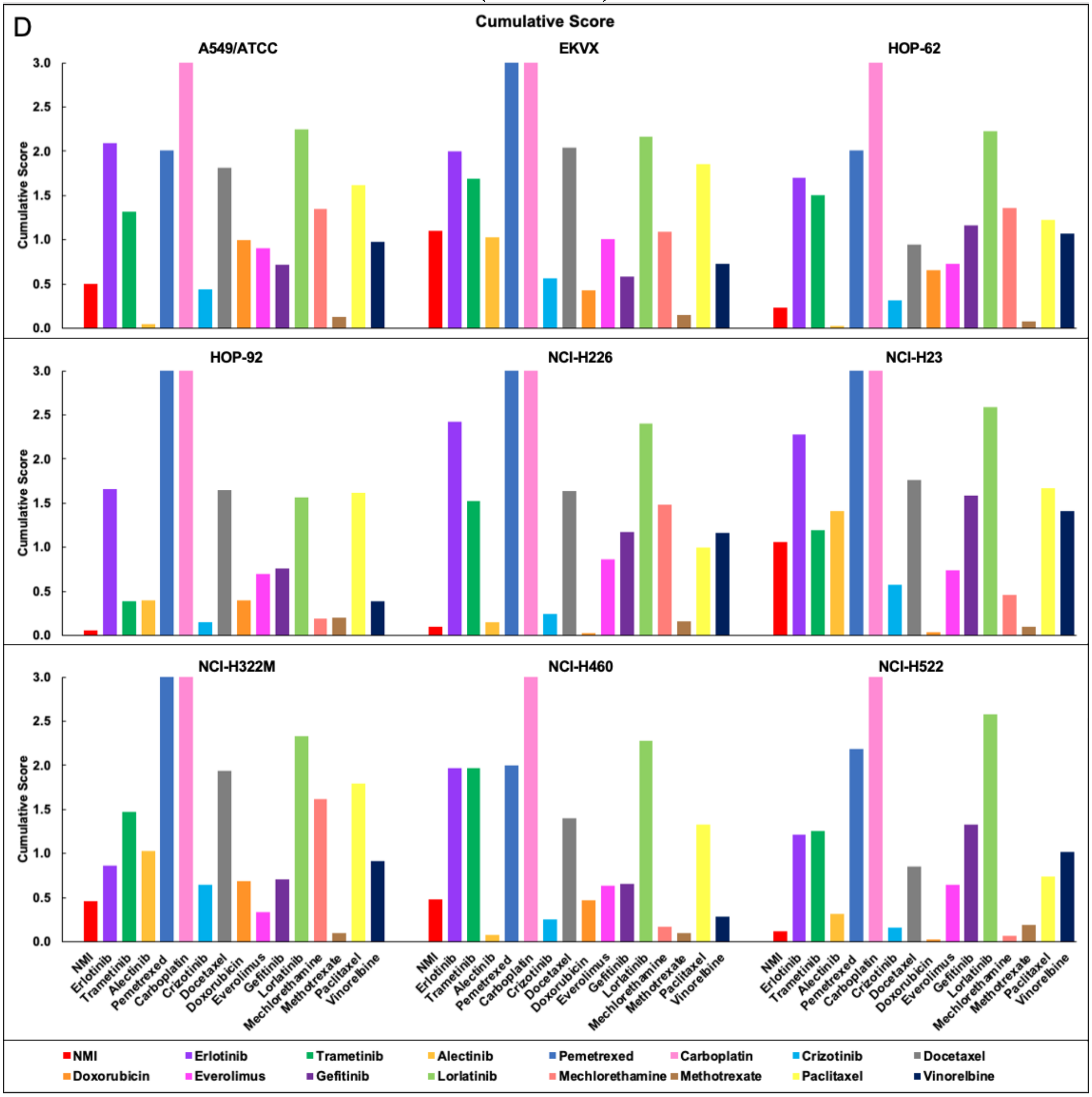

Figure S2 The comparison of (A)GI_{50}, (B) TGI, (C) LC_{50}, (D) cumulative score of NMI to FDA-approved NSCLC drugs. NMI shows higher potency to NSCLC cell lines than most FDA-approved NSCLC cancer drugs. Y-axis indicates concentration of GI_{50}, TGI, LC_{50} (0 μM to 100 μM), and cumulative score (0.0 to 3.0). Drugs are color-coded: NMI (■); Erlotinib (■); Trametinib (■); Alectinib (■); Pemetrexed (■); Carboplatin (■); Crizotinib (■); Docetaxel (■); Doxorubicin (■); Everolimus (■); Gefitinib (■); Lorlatinib (■), Mechlorethamine (■); Methotrexate (■); Paclitaxel (■); Vinorelbine (■).

Table S3. The COMPARE results between NMI and FDA-approved CNS cancer, prostate cancer, and NSCLC drugs. All PCC values lower than 0.8, NMI has unique mechanisms than FDA-approved CNS, prostate, and NSCLC cancer drugs. Table of COMPARE results of Figure 5 and Figure S3. Exact values generated by COMPARE algorithm are provided.

CNS Cancer				
**Drug**	**NSC**	**PCC-GI50**	**PCC-TGI**	**PCC-LC50**
Temozolomide	362856	0.00	-0.08	No data (StdDev = 0)
Lomustine	79037	0.48	0.04	0.23
Carmustine	409962	0.31	-0.04	0.27
Everolimus	733504	0.02	0.13	0.51
**Prostate Cancer**				
**Drug**	**NSC**	**PCC-GI50**	**PCC-TGI**	**PCC-LC50**
Rucaparib	756644	No data	No data	No data
Enzalutamide	755605	-0.15	0.38	0.29
Abiraterone Acetate	749227	0.07	0.14	0.17
Cabazitaxel	761432	0.13	0.11	0.24
Docetaxel	628503	0.26	0.19	0.15
Leuprolide Acetate	746847	0.41	No data (StdDev = 0)	No data (StdDev = 0)
Mitoxantrone	758450	0.25	0.24	0.42
Olaparib	753686	0.09	0.07	No data (StdDev = 0)
**Non-Small Cell Lung Cancer**				
**Drug**	**NSC**	**PCC-GI50**	**PCC-TGI**	**PCC-LC50**
Erlotinib	718781	-0.27	0.09	0.03
Trametinib	758246	-0.14	0.26	0.32
Alectinib	764040	0.23	0.15	0.21
Pemetrexed	755384	0.03	No data (StdDev = 0)	No data (StdDev = 0)
Carboplatin	241240	No data (StdDev = 0)	No data (StdDev = 0)	No data (StdDev = 0)
Crizotinib	756645	0.44	0.46	0.34
Docetaxel	628503	0.26	0.19	0.15
Doxorubicin	123127	0.58	0.37	0.45
Everolimus	733504	0.02	0.13	0.51
Gefitinib	715055	-0.04	0.03	0.22
Lorlatinib	780108	0.1	0.01	No data (StdDev = 0)
Mechlorethamine	762	0.06	0.25	0.34
Methotrexate	740	-0.01	0.06	No data (StdDev = 0)
Paclitaxel	125973	0.38	0.24	0.44
Vinorelbine	608210	0.59	0.16	0.24

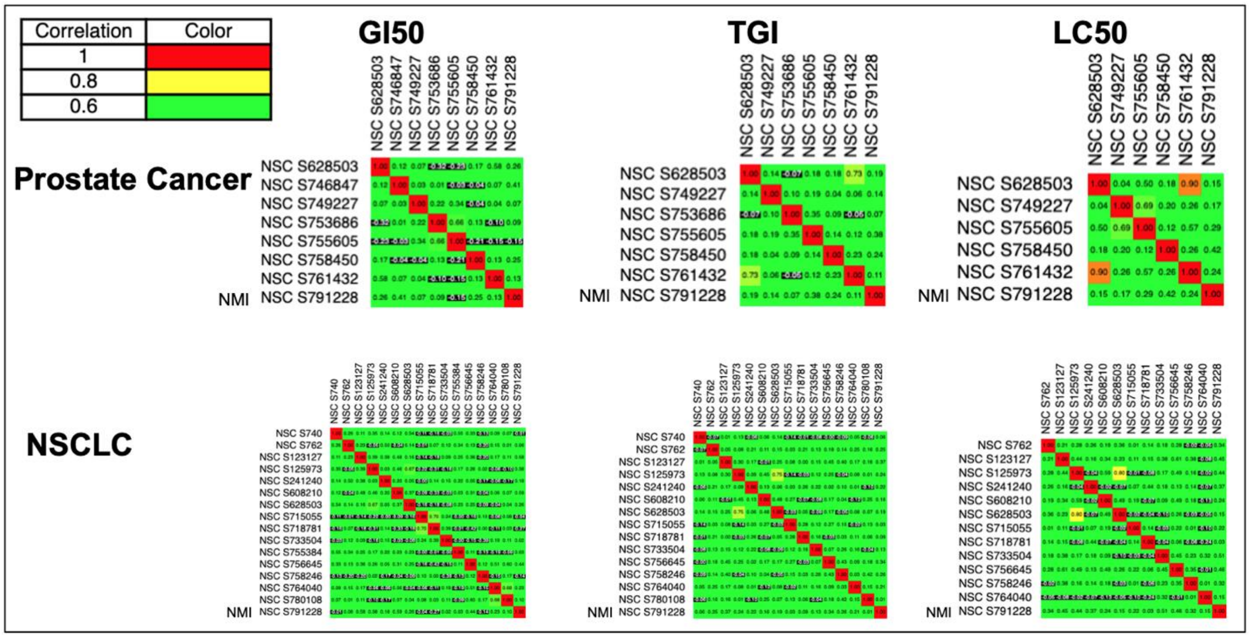

Figure S3. Comparison of the mechanism of NMI to FDA-approved prostate cancer and NSCLC drugs by COMPARE plots. NMI has unique mechanism compared to FDA-approved prostate and NSCLC cancer drugs. High PCC (Pearson Pairwise correlation coefficient), > 0.8 shown in yellow (■), orange (■), or red (■) in the matrix COMPARE figure, indicates these 2 drugs have similar mechanisms for cancer therapy.

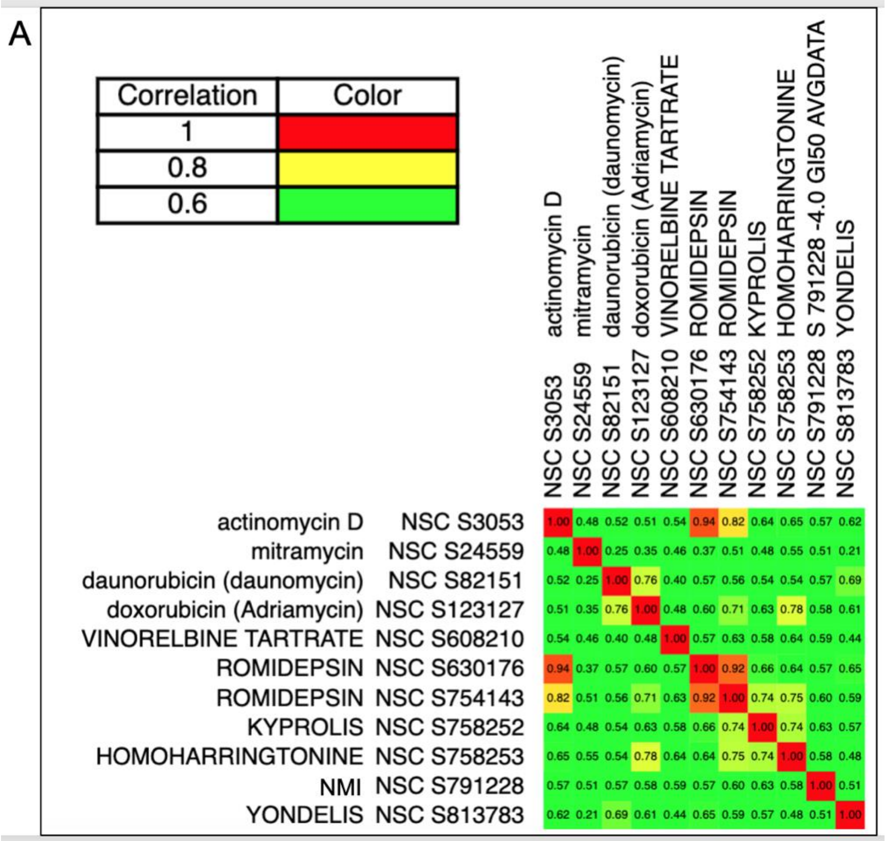

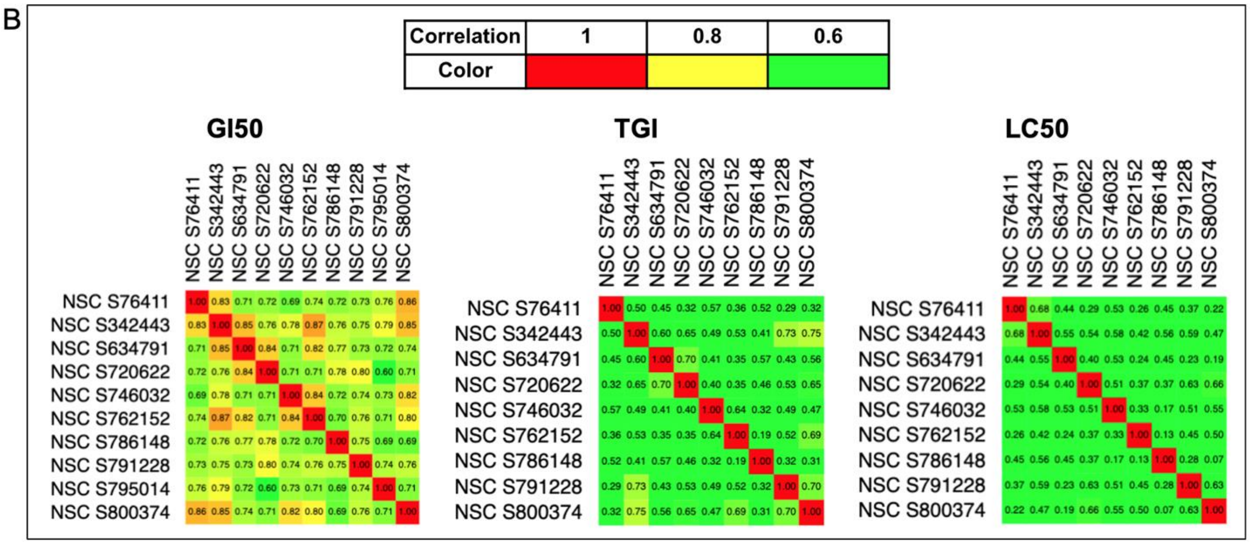

Figure S4. COMPARE plots of NMI (NSC 791228) to (A)marketed, (B)unmarked drugs in all cancers (top 10) PCC were shown. The result showed NMI has unique mechanism compared to all marketed anti-cancer drugs ; NMI might have similar mechanism with some unmarketed drugs. The Pairwise Pearson Correlation Coefficient (PCC) was determined by searching PUBLIC COMPARE Web Site Navigation for all marketed or unmarketed drugs in all different cancers in NCI60 database.

Table S4. Table of the COMPARE results of Figure S4A, with the drug names and their mechanisms.

NSC No.	Drug Name	PCC-GI50	Mechanism of Action
758252	Carfilzomib (Kyprolis)	0.63	Selective Proteasome Inhibitor
754143	Romidepsin	0.60	Histone Deacetylase Inhibitor
608210	Vinorelbine Tartrate	0.59	Antimicrotubular Antineoplastic Agent
123127	Doxorubicin	0.58	Topoisomerase Inhibitor
758253	Homoharringtonine	0.58	Protein Synthesis Inhibitor
3053	Actinomycin D	0.57	Transcription Inhibitor
82151	Daunorubicin	0.57	Topoisomerase Inhibitor
630176	Romidepsin	0.57	Histone Deacetylase Inhibitor
24559	Mitramycin	0.51	RNA Synthesis Inhibitor
813783	Trabectedin (Yondelis)	0.51	Alkylating Drug
791228	NMI	1.00	MAO A Inhibition; HIF1α Stabilization
